# Effects of blood flow restriction (BFR) with resistance exercise on musculoskeletal health in older adults: a narrative review

**DOI:** 10.1186/s11556-022-00294-0

**Published:** 2022-06-20

**Authors:** Zi Xiang Lim, Jorming Goh

**Affiliations:** 1 Healthy Longevity Translational Research Program, Yong Loo Lin School of Medicine, National University of Singapore (NUS), Singapore, 117456 Singapore; 2 Department of Biochemistry, Yong Loo Lin School of Medicine, National University of Singapore (NUS), Singapore, 117456 Singapore; 3 Centre for Healthy Longevity, National University Health System (NUHS), Singapore, 117456 Singapore; 4 Department of Physiology, Yong Loo Lin School of Medicine, National University of Singapore (NUS), Singapore, 117456 Singapore

**Keywords:** Skeletal muscle hypertrophy, Sarcopenia, Hypoxia, Hyperaemic reperfusion, Low intensity exercise

## Abstract

**Background:**

Aging leads to a number of structural and physiological deficits such as loss of muscle mass and strength. Strength training at ~ 70% of 1 repetition max (RM) is recommended to prevent age-related loss of muscle mass and strength. However, most older adults may not be able to perform 70% of 1RM or higher intensity. An alternative exercise training program combining low intensity resistance exercise with blood flow restriction (BFR) can result in similar acute and chronic benefits to skeletal muscles in older adults.

**Main body and short conclusion:**

The potential mechanisms involved are discussed, and include reactive hyperaemia, metabolic stress, and hypoxia. Key issues and safety with the use of BFR in older adults, especially those with chronic conditions are also discussed. Although there has been no reported evidence to suggest that BFR elevates the risk of clinical complications any more than high intensity exercise, it is recommended for individuals to be medically cleared of any cardiovascular risks, prior to engaging in BFR exercise.

## Background

Aging is characterised by a number of structural and physiological deficits, for instance, increased arterial stiffness leads to decreased vascular compliance [1] and elevates the risk for developing cardiovascular and metabolic disease. Furthermore, significant loss in skeletal muscle mass and strength starts around the 4th decade in life [2] and accelerates thereafter in men and women [3]. Age-related loss of muscle mass (sarcopenia) and strength contributes to functional decline, thereby increasing the prevalence of frailty, disability, falls and mortality in older adults [4, 5]. The estimated global prevalence of sarcopenia (ranged from 10 to 27%, depending on definition used) [6], imposes significant challenges on the global healthcare systems [7–9]. As recommended by the sarcopenia committees around the world, exercise is beneficial to prevent/reduce sarcopenia. It is important for the older population to exercise, to maintain skeletal muscle mass [10, 11].

The American College of Sports Medicine (ACSM) recommends strength training to prevent age-related loss of muscle mass and strength, with prescribed intensities ranging from loads of 65 to 75% of 1 repetition max (RM) for older adults [12]. Several studies have shown that exercising at intensities above 70% of 1RM improves muscle size and strength in older adults [13, 14]. Performing resistance exercise above 70% of 1RM also stimulated muscle protein synthesis, satellite cell activity, and decreased proteolysis when compared with low-intensity resistance exercise [15, 16].

Muscle hypertrophy can be achieved by manipulating exercise intensity (load, repetitions), duration, or both. Some investigators have suggested that the training load may be redundant; exercising to failure with low (~ 30% 1RM) or high loads (~ 80% 1 RM) resulted in similar rates of muscle hypertrophy [17, 18]. A disadvantage in using low loads (~ 30% 1RM) is the longer duration required to reach muscular fatigue. To economize exercise time, it may be prudent to increase the intensity of exercise. Resistance training with heavier loads is necessary to maximize the development of muscle mass and strength [13, 19].

**Fig. 1:**
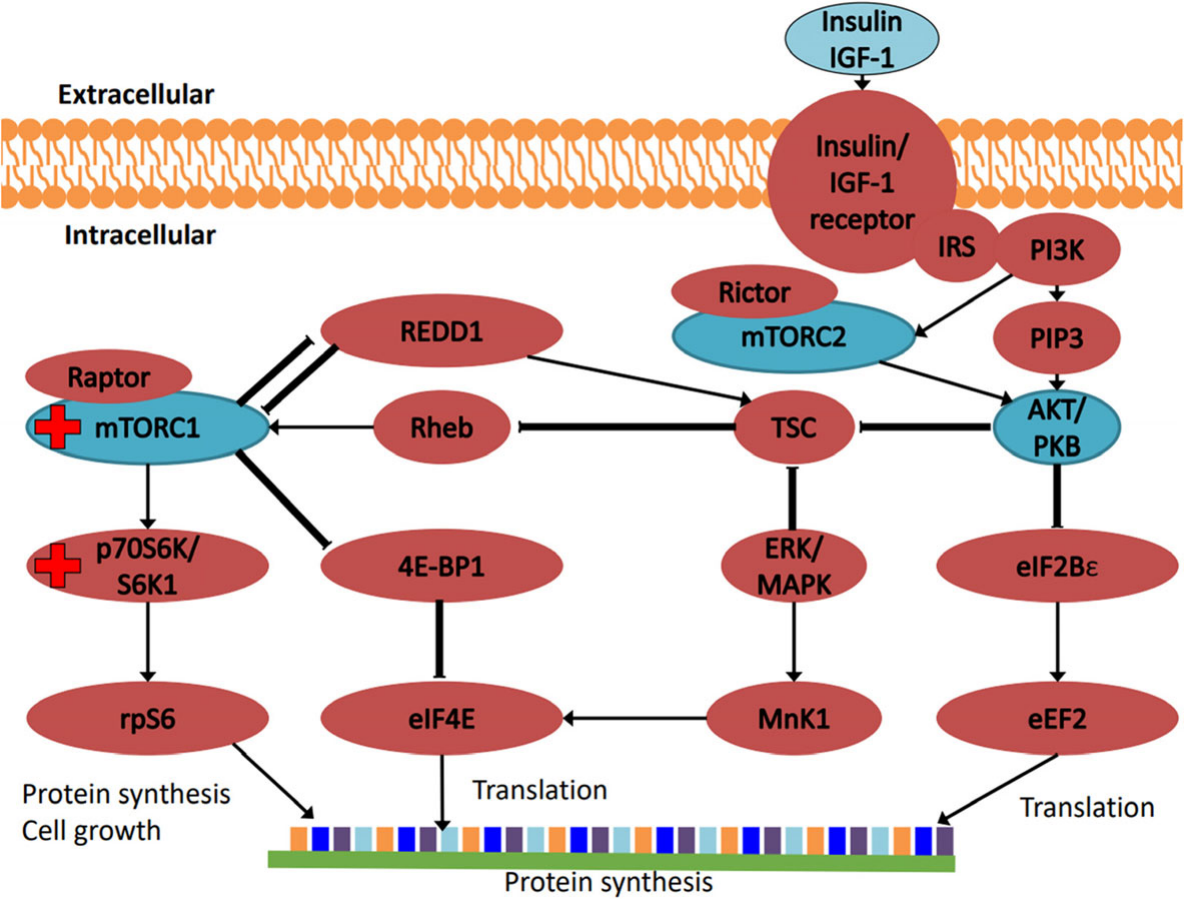
AKT-mTOR signalling pathways in skeletal muscle for protein synthesis. Red “+” sign indicates hyperphosphorylation of protein at baseline with aging. 4E-BP1: 4E binding protein 1; AKT: protein kinase B; eEF2: Eukaryotic elongation factor 2; eIF2Bε: eukaryotic translation initiation factor 4E; ERK/MAPK: extra-cellular signal-regulated kinase; IGF-I: insulin-like growth factor 1; IRS: insulin receptor substrate; MnK1: mitogen-activated protein kinase-interacting kinase 1; mTORC1/2: mammalian target of rapamycin complex 1/2; PI3K: phosphatidylinositol-3 kinase; PIP3: phosphatidylinositol-3,4,5-triphosphate; REDD1: regulated in development and DNA damage responses 1; Rheb: ras-homolog enriched in brain; rpS6: ribosomal protein S6 (S6); S6K1; ribosomal protein S6 kinase beta-1 (p70S6 kinase 1); TSC: tuberous sclerosis complex

High-intensity resistance exercise stimulates the molecular pathways that regulate protein synthesis/hypertrophy. Skeletal muscle from young adults performing high-intensity resistance exercise (70% 1RM) showed an activation of the mammalian target of rapamycin (mTOR) signalling pathway (Fig. 1): protein kinase B (AKT); ~ 50%, mTOR; ~ 100%, ribosomal protein S6 kinase beta-1 (S6K1); ~ 150%, 4E binding protein 1 (4E-BP1); ~ 50%, extracellular signal-regulated kinase 1/2 (ERK1/2); ~ 300%, ribosomal protein S6 (S6; ~ 200%), and increased protein synthesis (~ 60%) 3 to 24 hours post-exercise [20]. However, older adults performing the same resistance exercise had reduced extent of, or did not achieve significant changes in muscle protein synthesis, or phosphorylation of intramuscular proteins in the mTOR pathway [20], suggesting that aging impairs the anabolic response to acute high-intensity resistance exercise. While inconclusive, the impaired anabolic response in older adults has been suggested to be associated with basal hyperphosphorylation of S6K1 [21] and basal mTOR complex 1 (mTORC1; Fig. 1) [22]. Partial inhibition of mTORC1, using Everolimus, a rapamycin-mimetic (rapalog), counteracted age-associated sarcopenia in aged rat models [23]. Hyperphosphorylation of these intramuscular signalling proteins could reduce AKT phosphorylation and degradation of Insulin receptor substrate 1 (IRS-1) [24]. Therefore, it is possible that chronic phosphorylation of the mTORC1/S6K1 pathway impairs the age-related anabolic response. In support of this viewpoint, it had been demonstrated that mTORC1 is hyperphosphorylated in skeletal muscles of sarcopenic rats [23].

A possible solution to overcome the impaired anabolic response in older individuals is to increase the volume of exercise training. Doubling resistance exercise volume, by increasing from 3 to 6 sets of 40% 1RM and 75% 1RM, upregulated intramuscular protein expression (S6K1; ~ 100% and ~ 75% respectively) and muscle protein synthesis (~ 300% and ~ 100% respectively) in older adults, but was not further enhanced in young adults [25]. However, high-intensity load and increased training volume in resistance or aerobic training programs are impractical for older adults with chronic medical conditions, or in older, deconditioned adults. This is primarily because a major barrier for older adults to commit to exercise programs is the perceived time constraint [26]. Therefore, an alternative solution would be needed to recapitulate the benefits of exercise in the older adults.

An alternative exercise training method that combines blood flow restriction (BFR) with low-intensity exercise, both resistance and aerobic, can result in positive physiological adaptations akin to performing high-intensity exercise. For instance, combining BFR with low-intensity resistance training increased intramuscular signaling pathways and the rate of muscle protein synthesis in younger [27] and older adults [28], as compared with low-intensity resistance training alone. In addition, combining BFR with low-intensity exercise was as effective as high-intensity exercise in increasing muscle mass and strength [29–32].

Given that there may be different mechanisms involved with the use of BFR in older adults as compared to younger adults, the purpose of this narrative review is to summarise the short- and long-term effects of combining BFR with resistance exercise on physiological and molecular responses in older adults. The adaptations and potential mechanisms with the use of BFR alone, or with exercise in older adults will be discussed in the next section. Finally, key issues and safety of the use of BFR in older adults will be discussed.

### History and origin of BFR

The origins of BFR can be traced as far back as 1966, where Dr. Yoshiaki Sato’s leg became numb after sitting at a Buddhist memorial in a kneeling position [33]. He then started to massage his calf and noticed the swelling and discomfort was similar to performing calf-raise exercises [33]. Dr. Sato then experimented and eventually developed and patented the KAATSU training method [33], which combines BFR with low-intensity resistance exercise (~ 20–50% 1RM). The KAATSU method typically involves applying a tourniquet that occludes up to 200 mmHg of systolic blood pressure (SBP), while performing low-intensity resistance exercise (Table 1).

**Table 1:** Acute resistance exercise with BFR in older adults

Article	Subject characteristics	Exercise protocol	BFR method	Side effects	Research outcomes
[92]	14♀, (45 ± 9.9 yo) Hypertensive & sedentary	30% 1RM, bilateral knee extension 3 × 15 reps 45 sec rest between sets	80% of arterial pressure occlusion	Not mentioned	SBP up to 60 min post-exercise 13.2% **↓** HR **↔** DBP **↔**
[28]	7♂, (70 ± 2 yo) Healthy, nonclinical Physically active but no structured training	20% 1RM, bilateral knee extension 30, 15,15, 15 reps 30 sec rest between sets	200 mmHg	Reported discomfort comparable to high-intensity resistance exercise. No side effects reported up to 1-week post-exercise	Leg circumference 52% **↑** **Blood markers** Blood lactate 175% **↑** 15 min post-exercise, back to baseline 60 min post-exercise. Cortisol ~ 1100% **↑** post-exercise, back to baseline 3 hr. post-exercise GH ~ 400% **↑** 15 min post-exercise, back to baseline 45 min post-exercise IL-6 **↔** IGF-I **↔** D-dimer **↔** Blood glucose **↔** **Intramuscular** Muscle protein synthesis ~ 50% **↑** mTOR Ser2448 ~ 30% **↑** 3 hr. post-exercise S6K1 Thr389 ~ 300% **↑** 3 hr. post-exercise S6 Ser235/235 ~ 1000% **↑** & 1800% **↑**, 1 & 3 hr. post-exercise S6 Ser 240/244 ~ 500% **↑** 3 hr. post-exercise MnK1 Thr197/202 ~ 200% **↑** 1 hr. post-exercise AKT Thr308 ~ 72% **↑** 3 hr. post-exercise 4E-BP1, eEF2, eIF2Bε **↔** REDD1 gene expression 41% **↓** 3 hr. post-exercise REDD2 gene expression 42% **↓** 3 hr. post-exercise REDD1 protein **↔** AMPKα Thr172 **↔** HSP70 **↔** FAK Tyr576/577 **↔** FOXO3a Ser253 **↔**
[38]	7♂, (71.0 ± 6.5 yo) Healthy, nonclinical Performs regular aerobic exercise, but not resistance exercise	20% 1RM, unilateral knee extension 5 sets (30, 9, 6, 5, 4 reps) 30 sec rest between sets	~ 110 mmHg	Not mentioned	GH ~ 220% **↑** 30 min post-exercise Vascular endothelial growth factor ~ 250% **↑** up to 2 hour post-exercise IL-6 ~ 15% **↑** 1- & 2-hour post-exercise Cortisol **↔** IGF-1 **↔**
[42]	18♀, (67.0 ± 1.7 yo) Hypertensive & sedentary	20% 1RM, bilateral knee extension 3 × 10 reps 1 min rest between sets	80% of arterial pressure occlusion	Not mentioned	**During exercise** SBP ~ 60.0% **↑** DBP ~ 62.6% **↑** HR ~ 22.1% **↑** SVR ~ 66.5% **↑** SV **↔** CO **↔** **Post-exercise** SBP ~ 2.2% **↓** DBP ~ 2.4% **↓** HR ~ 8.1% **↓** SVR **↔** SV ~ 30.7% **↓** CO ~ 27.9% **↓** Lactate ~ 28.6% **↑**
[101]	12♀, (57 ± 7 yo) Hypertensive & sedentary	20% 1RM, leg press 3 × 15 reps 30 sec rest between sets	100% of arterial pressure occlusion	Not mentioned	**During exercise** SBP ~ 62.2% **↑** DBP ~ 69.6% **↑** HR ~ 58.3% **↑** CO ~ 20% **↑** SVR only in 2nd & 3rd set ~ 42.0% **↑** **During rest intervals** SBP ~ 35.9% **↑** DBP ~ 15.1% **↑** HR ~ 14.5% **↑** CO ~ 17.8% **↓** SVR ~ 54.6% **↑**
[43]	13♂, (70 ± 5 yo) Healthy, nonclinical & sedentary	20% 1RM, leg press 30, 15, 15, 15 reps 1 min rest between sets 2 sec concentric, 2 sec eccentric	60% of arterial pressure occlusion	Not mentioned	**Leg press trial** MAP & SBP **↑** Lactate **↑** *BFR and non-BFR trial performed on same day with 20–40 min rest between exercises.

**↑**Indicates significantly increased

**↓**Indicates significantly decreased

**↔**Indicates no significant difference

4E-BP1: 4E binding protein 1; AKT: protein kinase B; AMPKα: 5′ adenosine monophosphate-activated protein kinase; eEF2: Eukaryotic elongation factor 2; eIF2Bε: eukaryotic translation initiation factor 4E; ERK/MAPK: extra-cellular signal-regulated kinase; FAK: Focal adhesion kinase; FOXO3a: Forkhead box transcription factors; HSP70: Heat stress protein 70; IGF-I: insulin-like growth factor 1; IRS: insulin receptor substrate; MnK1: mitogen-activated protein kinase-interacting kinase 1; mTORC1/2: mammalian target of rapamycin complex 1/2; PI3K: phosphatidylinositol-3 kinase; PIP3: phosphatidylinositol-3,4,5-triphosphate; REDD1: regulated in development and DNA damage responses 1; Rheb: ras-homolog enriched in brain; rpS6: ribosomal protein S6 (S6); S6K1; ribosomal protein S6 kinase beta-1 (p70S6 kinase 1); TSC: tuberous sclerosis complex; CO: Cardiac output; DBP: diastolic blood pressure; GH: growth hormone; HR: heart rate; IL-6: interleukin-6; MAP: mean arterial pressure SBP: systolic blood pressure; SV: stroke volume; SVR: systemic vascular resistance

### Benefits of BFR without exercise

Without incorporating exercise, BFR alone reduced disuse atrophy by 9.4% and 9.2% in knee extensors and flexors respectively, compared with control (limb immobilization) [34]. Further, BFR reduced and delayed skeletal muscle atrophy in young healthy adults immobilised with casts after injury [34–36], as well as in older adults with chronic disease [37]. These studies suggest that BFR-induced hypoxia and/or hyperaemic reperfusion may preserve muscle mass in age-associated sarcopenia. Apart from improving disuse atrophy, BFR was also effective in mitigating strength reduction during limb immobilisation [35, 36]. Given the paucity of studies investigating the effects of BFR in physiological responses in older adults, future studies should explore whether BFR mitigates changes in muscle strength and size differently between young adults and older adults.

### Acute BFR with resistance exercise in older adults

Combining BFR with a single session of resistance exercise in older men increased phosphorylation of proteins involved in skeletal muscle anabolism (mTORC1; ~ 30%, S6K1; 300%, S6 Ser235/236; ~ 1800%, S6 Ser240/244; ~ 500%), and muscle protein synthesis (~ 56%) immediately post-exercise [28]. The concentration of anabolic hormones such as growth hormone (GH), was also increased in systemic circulation 15–30 min after combined BFR and resistance exercise, and returned to resting concentrations 45 mins post-exercise [28]. It is important to note that the increase in systemic concentrations of GH was observed post-BFR with low-intensity resistance exercise but not with low-intensity resistance exercise alone [38]. The reduced systemic GH concentrations in normal aging is associated with reduced muscle mass and strength and slower muscle protein synthesis [39]. Administration of GH in older adults increased muscle protein synthesis and muscle growth, which may be mediated through the insulin-like growth factor 1 (IGF-1) signalling pathway [40, 41]. BFR with resistance exercise also elevated other blood biomarkers such as cortisol [28], lactate [28, 42, 43] and interleukin-6 (IL-6) [38], which are related to exercise-induced metabolic stress and contribute to skeletal muscle adaptations [44, 45]. An elevation of these biomarkers in systemic circulation during a combined session of BFR with resistance exercise would suggest that this exercise mode is sufficient for skeletal muscle adaptations.

### BFR with chronic resistance training in older adults

Chronic resistance exercise training improves musculoskeletal health in older adults [43]. Maintaining or enhancing skeletal muscle mass can slow the rate of functional decline with aging [46–48], given the strong evidence that regular participation in physical activity improves physical capacity and mobility, while reducing the risk of fall-related injuries by 32–40% in older adults [49].

In older adults, performing 3 to 12 weeks of BFR with low-intensity resistance exercise increased muscle mass and strength (Table 2), compared with low-intensity resistance exercise without BFR [28] or with sedentary controls [50, 51]. The increases in muscle cross-sectional area (CSA) [29, 31, 32, 52–55], leg press (muscle strength) [30, 54, 55] and leg extension (muscle strength) [29–32, 53–55] in older adults were similar when compared with resistance exercises incorporating heavier loads (≥ 70% 1RM) [29–32, 52–55]. In a different study, 12 weeks of combined BFR with resistance exercise resulted in increased muscle size and strength, relative to high-intensity resistance exercise alone [56]. In addition, BFR combined with resistance exercise training improved cardiovascular health in older participants, with decreases in mean arterial pressure (MAP; 11.6%), SBP (11.0%) and diastolic blood pressures (DBP; 12.1%) respectively [57]. Maximal aerobic exercise capacity (V̇O_2max_; ~ 10%) improved when participants underwent 12 weeks of BFR with low resistance exercise training [29, 31, 32]. It is important, however, to note that these studies included aerobic exercise training. Hence, synergistic V̇O_2max_ adaptations with aerobic training may have occurred. These physiological improvements from combining BFR with resistance exercise also led to improvements in physical function, as measured by walking speed and the chair stand test [50, 54, 58]. Therefore, low-intensity resistance exercise combined with BFR is a viable mode of exercise for older adults.

**Table 2:** Resistance training with BFR in older adults

Article	Subject characteristics	Exercise Frequency	Rest duration	Exercise Intensity	Number of sets x reps	Blood flow restriction method	Side effects	Research outcomes
[57]	8♀, (63.8 ± 11.6 yo) Hypertensive & sedentary	8 weeks, 2 x/ week Wrist flexion 1.5 sec concentric, 1.5 sec eccentric	30 sec between sets	30% 1RM	3 sets 12, 10, 8 reps	70% of arterial pressure occlusion	Not mentioned	SBP 11.0% **↓** DBP 12.1% **↓** MAP 11.6% **↓** HR, Cortisol and IL-6 **↔**
[54]	Mixed (5♂, 7♀), (76.5 (72.3–80.7) yo) Healthy, nonclinical & sedentary	12 weeks, 2 x/ week Leg curl Leg extension 3 sec concentric, 3 sec eccentric	60 sec rest between sets 3 min rest between exercises	30% 1RM Leg press 50% 1RM	1st week 1 set to failure 2nd week 2 sets to failure 3rd week onwards 3 sets to failure	150% of arterial pressure occlusion	Not mentioned	Quadriceps CSA 4.3% **↑** Leg extension strength (1RM) 24% **↑** Leg press strength (1RM) 12% **↑** Walking speed 4% **↑** Short Physical Performance Battery, Quality of life **↔** Leg curl strength (1RM) **↔**
[29]	Mixed (5♂, 6 ♀), (63.3 ± 4.1 yo) Healthy, nonclinical & sedentary	12 weeks, 4 x/ week **Concurrent training:** i) 2 days Endurance training (ET) ii) 2 days low-intensity resistance exercise with BFR	Not mentioned	1st 6 weeks 20% 1RM 7th week onwards 30% 1RM	1 set × 30 reps 3 sets × 15 reps	50% of arterial pressure occlusion	Not mentioned	Quadriceps CSA 7.3% **↑** Leg press strength (1RM) 35.4% **↑** V̇O_2peak_ 10.3% **↑** C reactive protein **↑** IL-6 25% **↑** IL-10 **↔**
[100]	Mixed (6♂,10♀), (67.2 ± 5.2 yo) Healthy, non-clinical & sedentary	12 weeks, 3 x/ week Leg press Leg extension Leg curl Calf flexion	Not mentioned	20% 1RM	To failure	Equation (pressure mm Hg = 0.5 (SBP) + 2 (thigh circumference) + 5)	21 were deemed related or possibly related to the study (6 BFR; 15 moderate intensity resistance training (MIRT)) The majority of these were related to knee pain (*n* = 14), & the BFR group had less of these reports (*n* = 3) than the MIRT group (*n* = 11) A total of five serious adverse events were observed (2 BFR; 3 MIRT), with only one (BFR group) deemed related or possibly related to the study	Knee extensor strength ~ + 18.1% Gait speed + 8.6% Short physical performance battery ~ + 0.1% Lean mass ~ + 0.5%
[30]	13♂, (55.9 ± 1.0 yo) Healthy, nonclinical & recreationally active	6 weeks 3 x/ week **Upper body** (latissimus pull down, shoulder press & biceps curl) 3 sets, 8 rep, 80% 1RM, followed by BFR Leg press & Leg extension for three sets	1 min rest between sets, 5–10 min rest between exercises	20% 1RM	30, 15, 15 reps	Start with 160 mmHg, Increase by 20 mmHg if rate of perceived exertion (RPE) was lower than 16	Not mentioned	Lat pulldown ~ 15.9% **↑** Shoulder press ~ 9.6% **↑** Bicep curl ~ 19.3% **↑** Leg press 19.3% **↑** Leg extension 19.1% **↑**
[31]	Mixed (*n* = 10), no mention of gender proportion, (64 ± 4 yo) Healthy, nonclinical & sedentary	12 weeks, 4x/ week **Concurrent training:** i) BFR 2 x/ week Leg press ii) ET 2 x/ week: walk/run 30–40 min, 50–80% V̇O_2peak_	60 sec rest between sets	1st 6 weeks 20% 1RM 30% 1RM for next 6 weeks	30, 15, 15, 15 reps	50% of arterial pressure occlusion	Not mentioned	Quadricep CSA 7.6% **↑** Leg press (1RM) 35.4% **↑** V̇O_2peak_ 10.3% **↑**
[102]	Case study (n = 1, ♂, 91 yo) Hypertensive Sarcopenic Cardiovascular disease Sedentary	8 weeks, 3 x/ week Treadmill walking, 30% heart rate reserve Elbow flexion Elbow extension Leg press Leg extension	1 min rest between sets and exercises	30% 1RM	3 sets of 10 RM. No report on the number of reps.	50% of arterial pressure occlusion	No clinical interference during exercise. No reports of discomfort or pain during low-intensity BFR	Lean mass − 3.6% Hand grip + 17.9% **Isokinetic strength** Peak torque extension + 4.6% Total work + 1.5% Work fatigue + 27.5%
[103]	Mixed (8♂, 2♀), (67 ± 3 yo) Healthy, nonclinical, physically active but not resistance-trained	4 weeks 3 x/ week Plantar flexion with one leg BFR; Control leg without BFR on same person	1 min rest between sets 3 min rest legs	25% 1RM	3 sets Exercise to failure in BFR limb Control limb follows similar reps	110 mmHg	100% free of injury or complications	Calf girth ~ 1.17% **↑** SBP **↔** DBP **↔** **Plantar flexor** 1RM ~ 13.5% **↑** MVC ~ 17.6% **↑** **Isokinetic torque** 0.52 N/m ~ 15.7% 1.05 N/m **↔** 2.09 N/m **↔** Resting limb blood flow **↔** Blood flow after 5 min circulatory occlusion ~ 38.9% **↑**
[32]	10♀, (64 ± 4 yo) Healthy, nonclinical & sedentary	12 weeks, 4 x/ week **Concurrent training:** i) 2 days Resistance training: Leg press ii) 2 days ET 50–80% V̇O_2peak_, 30–40 min	60 sec rest between sets	1st 6 weeks 20% 1RM 2nd 6 weeks 30% 1RM	30, 15, 15, 15 reps	50% of arterial pressure occlusion	Not mentioned	V̇O_2peak_ ~ 10.3% **↑** Leg press (1RM) ~ 35.4% **↑** Quadriceps CSA ~ 7.6% **↑**
[74]	Mixed (16♂, 4♀), (72 ± 4 yo) Healthy, nonclinical & sedentary	4 weeks, 3 x/ week Leg extension Leg press Rowing Chest press	30 sec between sets	20% 1RM	3 × 20 reps	100% of arterial pressure occlusion	Not mentioned	**Acute effects** HR 33.8% **↑** SBP ~ 38.4% **↑** DBP ~ 45.2% **↑** **Blood markers** Lactate ~ 500% **↑** NE ~ 55.0% **↑** VEGF ~ 42.3% **↑** GH ~ 244.0% **↑** EPI **↔** **Long-term effect** RHI ~ 16.7% **↑** vWF ~ 4% **↓** TM **↔** tcPO2 ~ 16.7% **↑** Muscle strength- leg extension (1RM) ~ 19.0% **↑** Muscle strength- leg press (1RM) ~ 11.3% **↑** Muscle strength- rowing (1RM) ~ 9.18% **↑** Muscle strength chest press (1RM) **↔**
[55]	Mixed (3♂, 2♀), (59 ± 2 yo) Postmenopausal, healthy & recreationally active	**Upper body exercises** (seated chest press, seated row, seated shoulder press) followed by lower body exercises (knee extension, knee flexion, hip flexion, hip extension)	30 sec between sets 30–120 sec between exercises	Resistance band estimated ~ 10–30% 1RM	30, 15, 15 reps	Upper body exercise with BFR 120 mmHg Lower body without BFR	Not mentioned	Chest press strength ~ 8.0% **↑** Seated row ~ 5.4% **↑** Shoulder press ~ 5.0% **↑** Leg press ~ 7.6% **↑** Right hip extension ~ 9.7% **↑** Left hip extension ~ 8.9% **↑** Right hip flexion ~ 27.3% **↑** Left hip flexion ~ 39.6% **↑** Right knee extension ~ 12.8% **↑** Left knee extension ~ 7.9% **↑** Right knee flexion ~ 22.1% **↑** Left knee flexion ~ 12.8% **↑** Muscle thickness pectoralis major 17.4% **↑** Other muscle thickness **↔**
[52]	Mixed (*n* = 8), no mention of gender proportion (65.0 ± 2.0 yo) Healthy, nonclinical & sedentary	12 weeks, 2 x/ week Leg press 2 sec concentric, 2 sec eccentric	1 min rest between sets	1st 6 weeks 20% 1RM 30% 1RM for following weeks	30, 15, 15, and 15 reps	50% of arterial pressure occlusion	Not mentioned	Leg press (1RM) *p* = 0.067, ~ 15.8% **↑** Muscle CSA quadriceps ~ 5.93% **↑**
[53]	Mixed (3♂, 2♀), (64 ± 3 yo) Healthy, nonclinical & physically active	12 weeks, 2 x/ week Leg press	1 min rest between sets	1st 6 weeks 20% 1RM 2nd 6 weeks 30% 1RM	30, 15, 15, 15 reps	50% of arterial pressure occlusion	Not mentioned	Leg press (1 RM) ^#^ **↑** Muscle CSA quadriceps ^#^ **↑** ^#^ The magnitude of increase in strength and size were not provided Enriched 159 gene ontology pathways as compared to control Enriched 2 gene ontology pathways as compared to HI
[50]	Mixed (3♂, 6♀), (71.3 ± 7.1 yo) Healthy, nonclinical & sedentary	12 weeks, 2 x/ week Leg press 1.3 sec concentric, 1.3 sec eccentric Knee extension 1.0 sec concentric, 1.0 sec eccentric	30 sec rest between sets 90 sec rest between exercises	20–30% 1RM	30, 20, 15, and 10 reps	full- first day set 120 mmHg, subsequently increase 10-20 mmHg until 270 mmHg	Not mentioned	Muscle CSA quadriceps 8.0% **↑** Muscle CSA adductors 6.5% **↑** Muscle CSA gluteus maximus 4.4% **↑** Leg extension (1RM) 26.1% **↑** Leg press (1RM) 33.4% **↑** Chair stand test (reps) 18.3% **↑** HR, blood pressure, FMD, CAVI, ABI, FDP, D-dimer, CK **↔**
[51]	Mixed (2♂, 7 ♀), (71.8 ± 6.2 yo) Healthy, nonclinical & sedentary	12 weeks, 2 x/ week Bilateral arm curl Triceps press down 1.2 sec concentric, 1.2 sec eccentric	30 sec rest between sets 90 sec rest between exercises	“Heavy (Green)” resistance band for men “Thin (Yellow)” resistance band for women	30, 15, 15, 15 reps	full- first day set 120 mmHg, subsequently increase 10-20 mmHg until 270 mmHg	Not mentioned	Muscle thickness elbow flexor ~ 17.6% **↑** Muscle thickness elbow extensor ~ 17.4% **↑** Muscle strength elbow flexor MVC ~ 7.8% **↑** Muscle strength elbow extensor MVC ~ 16.1% **↑** HR, blood pressure, CAVI, ABI **↔**
[56]	10♀, (70 ± 6 yo) Healthy, nonclinical & physically active but not resistance trained	12 weeks, 2 x/ week Squat 1.3 sec concentric, 1.3 sec eccentric Knee extension 1.0 sec concentric, 1.0 sec eccentric	30 sec rest between sets 90 sec rest between exercises	One gold resistance band for squat One black resistance band for knee extension Considered low-intensity bands as compared with bands used in HI protocol in the same study.	30, 15, 15, 15 reps	full- first day set 120 mmHg, subsequently increase 10–20 until 200 mmHg	Not mentioned	**Acute effect** Anterior mid-thigh 10.6% **↑** **Chronic effect** Quadricep CSA 6.9% **↑** Knee extension (MVC) 13.7% **↑** Leg press (1RM) 16.4% **↑** Knee extension (1RM) **↔** c-SBP, c-Aix, CAVI, and ABI **↔**
[67]	Mixed (11♂, 5 ♀), (55 ± 7 yo) Healthy, nonclinical & sedentary	6 weeks, 3 x/ week Lateral knee extension one limb with BFR one limb without BFR 1.5 sec concentric, 1.5 sec eccentric	1 min rest between sets	30% 1RM	Volitional fatigue	1st week 150 mmHg or 50% of arterial occlusion pressure (whichever was lower) 2nd week onwards 80% arterial occlusion pressure but no higher than 240 mmHg	Not mentioned	Knee extension 1RM ~ 10.9% **↑** Anterior thigh muscle thickness ~ 5.1% **↑** Lateral thigh muscle thickness ~ 4.0% **↑** Pulse wave velocity ~ 6.7% **↑** Cardiovascular vascular conductance **↔**
[104]	16 ♀, (64.7 ± 3.7 yo) Healthy, nonclinical & sedentary	14 weeks, 2 x/ week Wrist flexion 1.5 sec concentric, 1.5 sec eccentric	40 sec rest between sets	40% 1RM	3 × 15 reps	70% of arterial pressure occlusion	Not mentioned	Wrist flexion strength (1RM) 55.3% **↑** Quality of life **↑** Chair stand test **↔**

**↑** Indicates significantly increased

**↓** Indicates significantly decreased

**↔** Indicates no significant difference

# Indicates result details not provided in article

- Indicates decrease, without statistical analysis as they are case studies/case series

+ Indicates increase, without statistical analysis as they are case studies/case series

1RM: 1 repetition maximal; ABI: ankle-brachial pressure Index; c-Aix: central-augmented Index; c-SBP: central systolic blood pressure; CAVI: cardio-ankle vascular index; CK: creatine kinase; CSA: cross sectional area; DBP: diastolic blood pressure; EPI: epinephrine; FDP: fibrinogen degradation products; FMD; flow mediated dialation; GH: growth hormone; HR: heart rate; IL-6/10: interleukin-6/10; MAP: mean arterial pressure; NE: norepinephrine; RHI: reactive hyperaemia index; SBP: systolic blood pressure; SV: stroke volume; SVR: systemic vascular resistance; tcPO2: transcutaneous oxygen pressure; TM: thrombomodulin; TPR: total peripheral resistance; VEGF: vascular endothelial growth factor; V̇O_2peak_: peak volume of oxygen consumed per min vWF: von Willebrand factor

### Potential mechanisms of combining BFR with low-intensity resistance exercise in stimulating muscle hypertrophy in older adults

#### Intramuscular signaling pathways

Older adults have impaired anabolic responses to high-intensity resistance exercise training, which may partially be attributed to repression of specific molecular signaling involving muscle hypertrophy, for instance, chronic phosphorylation of basal S6K1 [21] and mTORC1 [22]. However, when older adults performed BFR with low-intensity resistance exercise, mTOR, S6K1, S6, mitogen-activated protein kinase-interacting kinase 1 (MnK1), AKT phosphorylation peaked 3 hours post-BFR exercise [28]. In addition, significant increase in muscle protein synthesis was reported [28]. The post-exercise peak in intramuscular signalling pathways and muscle protein synthesis was similar to young adults performing high-intensity resistance exercise [20]. However, a key difference was that skeletal muscle 4E-BP1 was phosphorylated after the BFR exercise in young adults [28] but not in older adults [28]. A depressed protein expression of 4E-BP1 may be relevant for older adults in staving off sarcopenia. In pre-clinical models, 24 month-old, 4E-BP1 knockout mice demonstrated enhanced protein synthesis in skeletal muscle, under basal and stimulated conditions, compared with age-matched wildtype controls [59]. Furthermore, the knockout mice also exhibited increased grip strength and muscle mass, compared with the age-matched wildtype controls. These observations suggest that phosphorylated 4E-BP1 may be a repressor for downstream protein translation and targeting this gene may be a potential prophylactic for treating sarcopenia.

The mechanisms responsible for the hypertrophic response following BFR exercise are still poorly understood, but many mechanisms have been proposed. The sub-section below will briefly discuss some proposed mechanisms as to how combining BFR with low-intensity exercise can have positive adaptations.

#### Reactive hyperemia (ischemia/reperfusion)

During BFR combined with resistance exercise, blood flow is restricted, creating an ischemic response. Immediately after exercise, the release of the occlusion increases blood flow to the muscles, resulting in reperfusion where blood flow is increased above pre-occlusion levels. Therefore, one of the proposed mechanisms is that reactive hyperemic response with BFR exercise is responsible for muscle hypertrophy. A research study by Gundermann and colleagues [60] showed that BFR resulted in elevated blood flow post-exercise, delivering nutrients (such as glucose and phenylalanine) to skeletal muscles. However, they also found that the enhanced blood flow and nutrient delivery was not the primary mechanism responsible for mTORC1 signaling and muscle protein synthesis after BFR exercise [60], indicating that reactive hyperemia is not the primary mechanism. It is likely that the hypertrophic response following BFR exercise involves other signaling pathways that are yet to be elucidated.

#### Metabolic stress

Metabolic stress has been reported to be as equally critical as mechanical tension for the induction of muscle growth [61–64]. To test this hypothesis, Goto and colleagues [65] compared 2 different rest periods, using volume- or intensity-matched resistance exercise, with one protocol having 30 sec rest between sets to minimize metabolite accumulation, while the other protocol did not have rest periods. Results showed that the concentration of blood lactate was significantly higher following the no-rest protocol when compared to the exercise with rest periods [65]. After 12 weeks of training with the same protocol, the protocol without rest resulted in an increase in muscle CSA, relative to pre-exercise, while there was no difference with the rest protocol [65]. This indicates an association between metabolic stress and muscle hypertrophy.

The degree of metabolic stress, such as lactate accumulation, was also observed during and post-BFR resistance exercise in older adults [28, 42, 43]. Indeed, lactate increases satellite cell activity and anabolic signal (phosphorylation of p70S6K and mTOR) for muscle hypertrophy in C2C12 muscle cells [66]. The potential for metabolic stress to stimulate muscle hypertrophy in older adults have been demonstrated by several studies using similar BFR (~above 50% AOP or up to 20 mmHg) with resistance exercise training (~ 20–50% 1RM) protocol over a period of time [29, 31, 32, 50–56, 67]. BFR with aerobic exercise also resulted in muscle hypertrophy in older adults [68–70], but to a smaller extent (~ 3%), compared with combined BFR resistance exercise (~ 4–17%).

#### Hypoxia

Hypoxia occurs when there is a decrease in oxygen tension. Hypoxia created by BFR may contribute to enhanced metabolic response following resistance exercise. A meta-analysis of nine eligible studies showed that hypoxia resistance training causes an increase in muscle size and strength [71]. BFR walking increased the release of hypoxic inducing factor 1 alpha (HIF-1α) [72], suggesting the potential for HIF-1α to be a stimulus for muscle hypertrophy. However, HIF-1α does not seem necessary for muscle development as the HIF-1α knockout experiment showed no effect on muscle development [73].

Exercising with BFR also stimulates vascular endothelial growth factor (VEGF) release [72, 74]. It was found that VEGF deletion in adult mouse skeletal muscle impaired the skeletal muscle contraction and hypertrophy adaptations [75]. VEGF plays an important role in muscle hypertrophy. The increase in VEGF following BFR may play a role, in part, to improved vascular endothelial function.

It is important to note that these studies were conducted in young adults and not in older adults. More research is required to establish how BFR exercise leads to muscle hypertrophy in older adults, and whether HIF-1α and VEGF are mechanistically involved.

### Key issues and safety with the use of BFR

Gender and age can have different prevalence and rates of decline in skeletal muscle mass and strength. However, most studies examining the effect of BFR on the elderly recruited a mixed population of older men and women. An example is sarcopenia, where the prevalence of sarcopenia was reported more frequently in women younger than 70 years, while this was more frequent in men older than 80 years [76, 77]. One key factor affecting this difference is menopause in older women. The reason could be due to the decline in systemic concentrations of estrogen as women enter menopause [78]. The low systemic concentration of estrogen is associated with accelerated loss of skeletal muscle mass and strength [79, 80]. Postmenopausal women who underwent estrogen replacement therapy had systemic concentrations of estrogen that are similar to young women; further, resistance exercise enhanced their sensitivity to anabolic responses such as muscle protein synthesis [81]. In contrast, postmenopausal women who did not undergo estrogen replacement therapy did not observe any anabolic effects such as muscle protein synthesis, from the same exercise [81]. This suggests that the level of estrogen in the blood in postmenopausal women can affect muscle mass. Another factor that affects sarcopenia due to gender difference is testosterone. Testosterone level can predict skeletal muscle mass in the older male population [82]. Testosterone level is also positively associated with muscle strength and function [83], and that testosterone treatment in old hypogonadal men increased handgrip strength [84] and leg strength [85]. While the role of testosterone in older women is limited, low levels of testosterone in older women are often associated with lower skeletal muscle mass and strength [86].

Research design can also underestimate the effects of BFR exercise training. One example is a study by Fahs and colleagues (2014), where the same individual trains their legs with different protocols. Training one leg with BFR and the other leg without BFR may result in a crossover effect within the individual. For example, biomarkers and signalling proteins may differ in the leg training with BFR from the leg without BFR. But since 2 different protocols were performed on the same individual, biomarkers and signalling proteins from both protocols will mix up systematically in the individual, underestimating the effect of both protocols. This may explain the difference in these results, as compared to other studies where participants perform the same training on both legs [56].

Another important factor affecting BFR exercise is the occlusion pressure of the cuff. Some researchers used a standard occlusion pressure across all participants, as such, researchers must take into account cuff width and cuff material [87, 88]. For example, wider cuffs restrict blood pressure at a lower pressure [87]. On the other hand, other researchers have used and recommended the occlusion pressure to be individualized to the percent arterial occlusion pressure (%AOP) during exercise [89, 90]. Using this method accounts for individual blood pressure, cuff width, cuff material and limb width. For example, larger limbs will require a greater cuff pressure to fully restrict arterial blood flow regardless of cuff width [87]. Using %AOP may seem to individualize pressure for all participants, but it requires specialized equipment to measure arterial pressure. Another factor to consider is whether to use a full or partial occlusion pressure, to balance between exercise effectiveness and injury prevention.

Occlusion pressure is an important consideration for clinical conditions such as hypertension and osteoporosis, especially for older adults with these clinical conditions. There are concerns that full occlusion with exercise may cause discomforts, increase the risk of injury and compliance in those with hypertension and osteoporosis, especially in older adults, while partial occlusion with exercise may not achieve the desired musculoskeletal adaptations/benefits [91]. As such, Ilett and colleagues [91] have recommended the cuff pressure of at least 60–80% arterial occlusion pressure for BFR exercise to be effective in young adults. While there is no common consensus, more research is required to further explore this area with the older population.

While there are concerns regarding the use of BFR training for older adults, especially those with chronic conditions, studies have shown that BFR training is safe. Araújo and colleagues [92], reported a hypotensive effect of leg extension exercises with BFR in hypertensive adult women 60 mins after exercise. It is important to take note that the authors used only 2 training sessions. On the other hand, Brand et al. (2013) evaluated the effects of strength training without occlusion and found similar results. They demonstrated that resistance exercise was effective in reducing systolic blood pressure and diastolic blood pressure. It is important to emphasize that Brand and colleagues had used a 48-week strength training program without BFR on hypertensive adults and noted no change in hypertension levels with no adverse effect during training sessions [93]. The effect of acute exercise reduction in blood pressure of hypertensive subjects may last for up to 13 hours [94], whereas the effect of chronic exercise on blood pressure reduction remains to be evaluated. This hypotensive effect after a session of the exercise was also observed in older adults with hypertension [95]. BFR with low-intensity exercise has been recommended for clinical populations, including hypertension due to the lower risk of injury [96] and was reported safe for hypertension individuals [97, 98]. The use of BFR exercise on individuals with hypertension has been reviewed as safe and effective in promoting cardiovascular and musculoskeletal health [97–99]. It is also important to note that there were a few cases of discomfort reported [28, 100] and only one study [100] reported knee pain and adverse side effect, out of the 24 studies looking at the effects of BFR with resistance exercise on older adults reported here. Since the number of studies using BFR on older adults remains small, it is recommended that older participants undergo medical clearance prior to participating in BFR training.

## Conclusions

The use of BFR with resistance exercise can be a viable and effective method for older adults to maintain musculoskeletal health. Many mechanisms underlying the musculoskeletal and cardiovascular adaptations have been suggested, but the extent to which they contribute is unclear and remains to be elucidated. Despite low mechanical stress from low-intensity exercise, when performed together with BFR, the combined approach can elicit adaptations similar to performing high-intensity exercise. While there are some concerns with the use of BFR on the older adults and clinical populations such as those with hypertension and osteoporosis, there has been no reported evidence to suggest that BFR elevates the risk of clinical complications any more than traditional high-intensity exercise modes. For the use of BFR in the older population, individuals are to be medically cleared of any cardiovascular risks.

## Data Availability

Not applicable.

## Abbreviations

CK: Creatine kinase
CO: Cardiac output
CSA: Cross sectional area
DBP: Diastolic blood pressure
GH: Growth hormone
IGF-I: Insulin-like growth factor 1
IL-6: Interleukin-6
MAP: Mean arterial pressure
mTOR: Mammalian target of rapamycin
SBP: Systolic blood pressure
